# Implementation and factors affecting the nursing process among nurses working in selected government hospitals in Southwest Ethiopia

**DOI:** 10.1186/s12912-020-00498-8

**Published:** 2020-11-10

**Authors:** Zerihun Adraro, Daniel Mengistu

**Affiliations:** 1 Department of Neonatal Nursing, Kotebe Metropolitan University, Addis Ababa, Ethiopia; 2 School of Nursing College of Health Science Addis Ababa University, Addis Ababa, Ethiopia

**Keywords:** Nursing process, Implementation of the nursing process, Southwest Ethiopia

## Abstract

**Background:**

The nursing process was initially adopted from the general system theory, and was developed and implemented in the field of education. There is a demand to implement the nursing process in practical care in every health institution, but the perception remains that it is time-consuming and impractical. If the nursing process is not valued and not used, nurses may continue to intervene on the basis of a medical diagnosis rather than on the basis of a rational nursing Process steps. In any of the steps, oversight or omission can result in less than optimal nursing care. The purpose of this study was to assess implementation and factors affecting the nursing process among nurses working in selected government hospitals in Southwest Ethiopia.

**Methods:**

An institution-based cross-sectional descriptive study was conducted from March 10 to April 1, 2015 in three hospitals in southwest Ethiopia using self-administered questionnaires. This study included a total of 138 nurses using simple random sampling. Data were classified, coded and entered into epidemiological information version 3.5.3, and exported to the statistical package for social science version 20 for analysis, descriptive statistics were used to describe the variables, bivariable and multivariable logistic regression were used to see the effect of each variable on the dependent variable.

**Result:**

The nursing process was found to be 73.9% implemented. Compared to a Bachelor of Science nurses’, the likelihood of implementing the nursing process was less likely among diploma nurses. Nurses working in administratively supported hospitals implemented the nursing process more compared to those without administrative support. The nursing process had been implemented higher by trained nurses compared to untrained nurses.

**Conclusion:**

The implementation of nursing process was good where; nearly seven in every ten nurses implemented the nursing process. Low educational qualification, lack of training, and non-supportive hospital administration were predictors of the nursing process implementation. The health service management, in collaboration with Ethiopian nursing/professional associations and international governmental and non-governmental organizations should give continuous on the job professional development education, and develop nursing practice guidelines.

**Supplementary Information:**

The online version contains supplementary material available at 10.1186/s12912-020-00498-8.

## Background

The nursing process is a structured and systematic process of providing goal-oriented and humanistic nursing care that is both efficient and effective [1]. It is a standard for the provision of individualized, ongoing nursing care through standardized nursing language. It helps improve the relationship of nurses with patients, uses available resources for patient care, and creates good communication between practicing nurses and nursing practice requires an efficient use of the nursing process and engages nurses in activities that enhance knowledge of the nursing process. Effective implementation of the nursing process improves quality of care and promotes the development of knowledge based on clinical practice [2–4].

The nursing process is coherent with the perspective of measuring results by benchmarking and prototyping, and encourages uniformity in practice [5]. Implementing the nursing process is key to the core of professional nursing practice and allows nurses to deliver quality nursing care within a systematic goal-directed framework [6, 7].

There is a demand to implement the nursing process in practical care in every health institution, in hospitals as well as in the community as a whole, but the perception remains that it is time-consuming and impractical. If the nursing process is not valued and not used, nurses may continue to intervene on the basis of a medical diagnosis rather than on the basis of a rational nursing assessment, planning, evaluation, record keeping and feedback. In any of the steps, oversight or omission can result in less than optimal nursing care. If the nursing process is not used, the question could be asked how nurses assume responsibility and accountability for the patient and how to assess the quality of nursing care? [3, 8–10].

The nursing process is generally accepted in most countries but it is not implemented consistently. A study at the Brazilian teaching hospital revealed that all steps had been used, but not consistently carried out; in Taiwan, nurses followed the nursing process sequences and documentation; in Brazil, 98.7% of cases, assessment was done; in 90% of cases, diagnosis was made; and in 74% of cases, planning was carried out; in Mexico, there were problems with the implementations of the nursing process [3, 8, 11, 12].

Most countries in Africa had adopted the nursing process: however, problems were found in its implementation in clinical setting. Nurses generally agreed on the benefits of the nursing process in a study conducted in four African countries: however, they did not use it commonly in practice [13]. A research in Nigeria showed 40.37% performed assessment, 13.7% performed diagnosis, 43.1% performed nursing plan and 2.7% performed assessment. While in Kenya, nurses were having trouble doing all the steps of the nursing process. Ethiopia’s government has focused on the quality of health services, and nursing process quality. However, the implementation of the nursing process remains constrained [14]. The nursing process was not introduced at all in Mekele, Ethiopia, in Addis Ababa hospitals, 52.1% of nurses implemented the nursing process, in Tigray region Ethiopia, 35% of nurses implemented the nursing process, in Debrmarkos and Finote-selam hospitals, Ethiopia, 37.1% of nurses implemented the nursing process, 32.7% in Arba-Minch Ethiopia, 42.1% nurses were implementing nursing process in Afar region [2, 15–21].

The implementation of the nursing process is hindered by several factors. Health care facility associated factors like organizational structures and facilities the environment of work place, non-proportional nurse to patient ratio, lack of training and motivating factors like salary high patient flow and scarcity of resources. The other is nurse related factor such as level of education, knowledge and skills of nurses, experience and ability to gather needed materials. Other factors hindering the implementation of the nursing process were the severity of cases and the patient cooperation [15, 16, 18, 19].

Nurses are the main health-care force in Ethiopia, and majority work in public health-care institutions and health-care services has been limited, quality of service has been poor, nurses face problems where their work has been underrepresented. They assume roles in various tasks, such as laboratory personnel, dentists, counselors and social workers, to cover the shortage of professionals in the field [15, 22, 23].

Although the implementation of the nursing process has been well investigated in many developed countries, it has only rarely been investigated in developing countries, including Ethiopia [3]; this study therefore evaluated the implementation and factors affecting the nursing process and could provide information to researchers, program managers and nursing stakeholders.

## Methods

### Study area

The study was conducted in three selected hospitals in southwest of southern nation nationalities and people regional state (SNNPRS) of Ethiopia. The southwest part is the farthest and marginalized area of the region. There are four hospitals in the area: Tercha hospital, Mizan Aman hospital, Gebretsadik Shawo hospital, and Teppi hospital. Only the three hospitals were selected because there is no any study conducted about the nursing process in the hospitals, and Tercha hospital was not chosen because there was one study conducted about the nursing process and also the pretest was conducted there to prevent diffusion of information. Mizan Aman Hospital is located in the town of Mizan Aman, 568 km southwest of Addis Ababa, established in 1968 and providing medical, surgical, pediatric and gynecological services with 122 beds and 81 nurses. Gebretsadik Shawo hospital is located in the town of Bonga, 460 km south-west of Addis Ababa. It was founded in 2006, and had 77 nurses. Teppi hospital is located in Teppi town, 614 km southwest of Addis Abba, which was established in 2014 and has 32 nurses.

### Study design and period

Hospital based cross-sectional quantitative descriptive study was conducted from March 10 to April 1, 2015. The source population was all nurses working at the three hospitals. Nurses in the three hospitals with a diploma / above level of education and served for 6 months or more were included.

### Sample size

The sample size was calculated using a single population proportion formula, taking the margin of error (d) as 5%, the confidence level of 95%, α = 0.05, the non-response rate of 10% and the nursing process implementation proportion (P) as 52.1% [15].

$$ n=\left( z\alpha /2\right)2\ p\ \left(1-P\right)/d2 $$

Where n is the minimum sample size, z is the normal standard distribution (z = 1,96) at a confidence level of 95% and α = 0,05, p is the prevalence / population proportion and d is the tolerable margin of error.

A total of 140 nurses were included in the study after considering the formula for finite population correction and 10% non-response rate. The correction formula was used because the calculated sample size is greater than the total population and the total population is less than ten thousand.

### Sampling procedure

The total sample size was allocated proportionally to the three hospitals based on the number of nurses working in each hospital i.e. 24 nurses for Teppi hospital, 60 nurses for Mizan Aman hospital and 56 nurses for Bonga hospital, and sampling frame was prepared for each hospital by receiving list of nurses from each hospital’s human resource department. Finally from the sampling frame 140 nurses were selected using simple random sampling after the purpose and procedure for data collection was clarified, and confidentiality and privacy were guaranteed.

### Data collection techniques and tools

Data was collected using self-administered questionnaires that were adapted and modified from various studies [2, 15, 16] and had three parts: sociodemographic issues, nursing process implementation, and factors affecting the nursing process (see additional file 1). To ensure the validity of tool, the questionnaire was presented to three nursing expert, checked it for double, confusing and leading questions, tool validity determined using content validity and also the instrument was pre-tested before final data collection. Cronbach’s alpha cofficent for nursing process implementation scale was 0.73. The tools included open as well as close-ended questions prepared in English. One supervisor and three Bsc nurses collected the data at the workplace of the nurses after they received 1 day’s training .

### Study variables

#### Dependent variable

Implementation of the nursing process.

#### Independent variables

Sociodemographic (age, sex, educational level, religion, year of service, marital status, ethnicity, an institution where education award is obtained and monthly salary), organizational factors such as non-supportive hospital administration, resource scarcity, shortage of staff training, time shortage, monitoring and evaluation.

### Operational definition

Implementation of nursing process: in this study nurses who responded yes to the question did you follow the steps of the nursing process during provision of care? Were considered as implemented.

### Data quality assurance

Before the actual data collection pre-test was done on 5% (seven) nurses working in Tercha hospital, which is not part of the main study, and some modification was made to the tool and procedure based on the pre-test findings. Collectors and supervisors were given training. The data was checked by the supervisor on each day of collection for completeness and consistency. Before entry in to the computer and again before analyzing; the principal investigator rechecked for missing values.

### Data processing and analysis

The data was first checked for completeness and consistency then categorized coded and entered into EP info version 3.5.3 and exported to SPSS version 20 for analysis. Univariate analysis such as percentage and frequency distribution was used. Bivariate analysis was used; variables with *P*-value < 0.2 from the binary logistic regression analysis were moved to multivariable logistic regression analysis in order to control the effect of confounders and to identify predictors of nursing process implementation. Significance of association was tested using 95% confidence level and *p* < 0.05.

## Result

One hundred thirty-eight of the 140 sampled respondents in the selected hospitals agreed and participated in the study making the response rate of 98.5%.

### Sociodemographic characteristics

Among the total respondents, 74 (53.6%) were males, 65 (47.1%) of respondents were in the age group of 25–29 years, 83 (60.1%) were single, and 83 (63%) were orthodox religion followers. Majority (34.8%) was from kaffa ethnic group, 63.8% were diploma nurses, and 60.1% of respondents had less than 5 years work experience. Majority (86.2%) was graduate of government institution, 55.1% had monthly salary between 1664 and 3245 (Table 1).

**Table 1:** sociodemographic characteristics of nurses in selected government hospitals in Southwest Ethiopia, 2015

Characteristics	Response	frequency	Percent
Sex	male	74	53.6
	female	64	46.4
Age	20–24	49	35.5
	25–29	65	47.1
	30–34	15	10.9
	≥ 35	9	6.5
Marital status	Single	83	60.1
	Married	55	39.9
Religion	Orthodox	87	63
	Protestant	35	25.4
	Muslim	13	9.4
	Catholic	3	2.2
Ethnicity	Kaffa	48	34.8
	Amhara	42	30.4
	Oromo	18	13
	Sheka	16	11.6
	Others^a^	14	10.2
Educational level	Diploma	88	63.8
	Bsc	50	36.2
Year of experience	< 5	83	60.1
	5–9	47	34.1
	≥ 10	8	5.8
Institution of educational award	Government	119	86.2
	private	19	13.8
Monthly salary	≤ 1663	55	39.8
	1664–3145	76	55.1
	≥3145	7	5.1

^a^*- Wolayita, Tigre, Gurage, Bench, Dizi, Silitte*

### Implementation of the nursing process

**Fig. 1:**
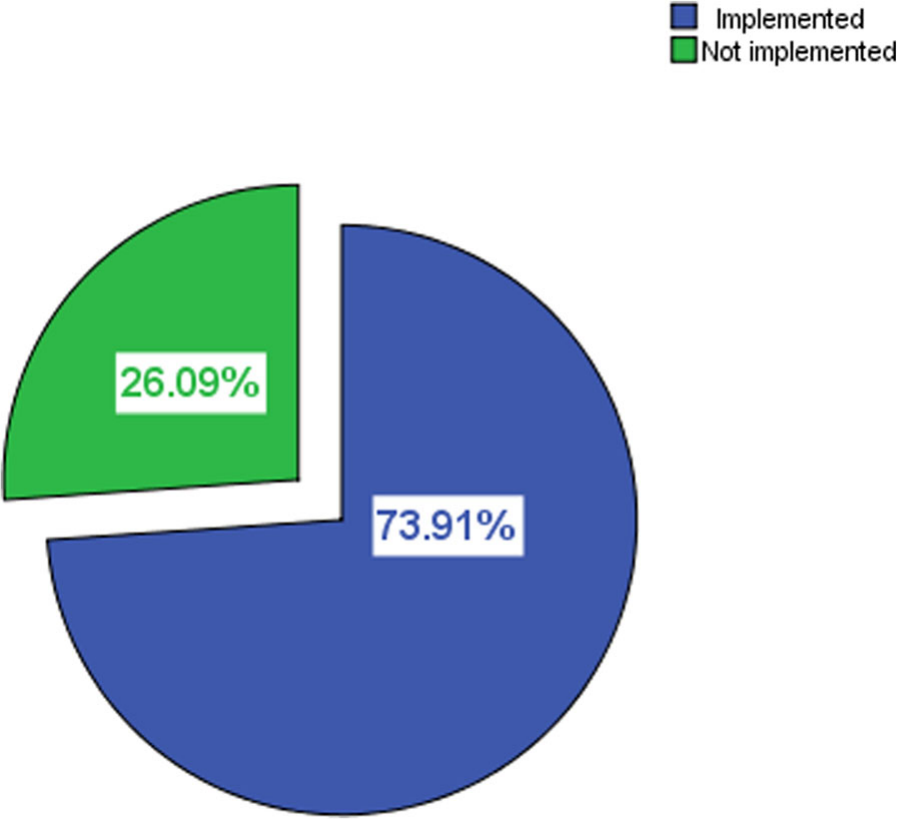
Percentage distribution of implementation of Nursing Process in selected government hospitals in Southwest Ethiopia, 2015

Concerning the implementation of the nursing process, the nursing process was implemented by 102 (73.9%) during care provision. With regards to the specific components implemented; 71.7% of those who implemented the nursing process developed nursing diagnosis, 71% developed care plan, 67.4% implemented the plan, 64.5% evaluated the intervention’s effectiveness and 70.3% documented their nursing activities. The actual diagnosis is 57.7%, 40.6% risk / potential, 5.8% likely, 4.3% health, and 4.3% collaborative were the type of diagnosis implemented (Fig. 1, and Table 2).

**Table 2:** Specific components of NP implemented in selected government hospitals, Southwest Ethiopia, 2015

Questions	Response	Frequency	Percent
Have you developed nursing diagnosis from your assessment?	Yes	99	71.7
	No	3	2.2
Type of nursing diagnosis you implemented^a^?	Actual	79	57.2
	Risk/potential	56	40.6
	Possible	8	5.8
	Wellness	6	4.3
	Collaborative	6	4.3
Have you been preparing care plan for your diagnosis?	Yes	98	71
	No	4	2.9
Have you been implementing care plan you have developed?	Yes	93	67.4
	No	9	6.5
Have you been evaluating effectiveness of your intervention?	Yes	89	64.5
	No	13	9.4
Have you been documenting your nursing care activities?	Yes	97	70.3
	No	5	3.6

^a^- more than one answer is possible, all questions in the table were asked only for those who implemented the nursing process (102 respondents)

### Organizational factors and facilities affecting implementation of the nursing process

Around two-third (68.1%) said that the hospital administration supported, 57.2% of responded time is not enough, and 53.6% of resources are not adequate. One-third (33.3%) of respondents said that the nurse-to-patient ratio was optimal for the nursing process and 44.2% had job training (Table 3).

**Table 3:** organizational factors and facilities affecting implementation of nursing process in selected government hospitals, Southwest Ethiopia, 2015

Questions	Response	Frequency	percent
**Does the hospital support implementation of NP?**	Yes	94	68.1
	No	44	31.9
**Is allocation of resource adequate to implement nursing process?**	Yes	74	46.4
	No	64	53.6
**Is allocated time sufficient to implement NP?**	Yes	59	42.8
	No	79	57.2
**Is the nurse-patient ratio optimal to implement NP?**	Yes	46	33.3
	No	02	66.7
**Is there monitoring and evaluation to implement NP?**	Yes	96	69.6
	No	42	30.4
**Are the salary and promotion motivating to implement NP?**	Yes	43	31.2
	No	95	68.8
**Have you got on job training about NP?**	Yes	61	42.2
	No	77	55.8
**Reasons not to receive training?**	Training not adjusted	61	44.2
	I am not willing	2	1.4

### Association of independent variables with the implementation of the nursing process

Results from the adjusted logistic regression analysis of factors affecting nursing process implementation after controlling for other factors had a statistically significant association between three factors. Diploma nurses were less likely to implement the nursing process compared to Bsc nurses, AOR = 0.36, 95% CI (0.14, 0.98), the likely hood of nurses working in hospitals promoting the implementation was 4.6 times higher than those who did not support the nursing process, AOR = 4.6, 95% CI, (1.87, 11.36), trained nurses are 3.8 times more likely to implement the nursing process compared to untrained nurses (AOR, 3,8, 95% CI, 1.47, 9.94). On binary logistic regression analysis monthly salary has significant association with NP implementation, but it has no statistically significant association in multivariable logistic regression analysis (Table 4).

**Table 4:** crude and adjusted odds ratio from logistic regression analysis of sociodemographic characteristics and organizational factors to the implementation of nursing process, Southwest Ethiopia, 2015

Variables		Implementation of NP		COR (95%, CI)	AOR (95%, CI)
		Yes No (%)	No No (%)		
Educational status	Diploma	60 (68.2)	28 (31.8)	0.41 (0.17, 0.98)	0.36 (0.14,0.98)^a^
	Bsc	42 (84)	8 (16)	1.00	
Monthly salary in ETB	≤ 1663	35 (63.6)	20 (36.4)	1.00	
	1664–3145	62 (81.6)	14 (18.4)	2.53 (1.14, 5.63)	2.31 (0.93, 5.71)
	≥3145	5 (71.4)	2 (28.5)	1.25 (0.25, 8.05)	1.22 (0.16, 9.04)
Administration support NP	Yes	78 (83)	16 (17)	4.06 (1.82, 9.05)	4.6 (1.87,11.36)^a^
	No	24 (54.5)	20 (45.5)	1.00	
Got training	Yes	52 (85.2)	9 (14.8)	3.12 (1.34,7.29)	3.8 (1.47, 9.94)^a^
	No	50 (64.9)	27 (35.1)	1.00	

^a^- had a statistically significant association for multivariate logistic regression

## Discussion

This study analyzed implementation and factors affecting the nursing process in selected government hospitals in Southwest Ethiopia. The nursing process was implemented by one hundred and two (73.9%) nurses during care provision. It was higher than a research carried out at Mekele zone hospitals, Ethiopia, where none of the nursing process steps were implemented [16]. The disparity might be due to the difference in the research setting in particular time that there might be progress in the nursing profession over the time period, resource and government commitment difference between regions. It might also be due to some difference in the tools used to collect the data and lack of nursing standard guidelines in the hospitals.

The finding was more higher when compared to different studies conducted in hospitals in Ethiopia; Addis Ababa Hospitals; 52.1%, Finote-selam and Debrmarkos hospitals; 37.1%, Northwest zone of Tigray region; 35%, Arba-Minch hospital; 32.7% and Afar region; 42.1% [15, 18–21]. The discrepancy could be due to the difference in the assessment tools used, sample size, nurses’ educational level and back ground, administration commitment and support from hospitals and regional health care system and facilities disparities. It might also be due to the methods used to operationalize the dependent variable. For instance Northwest zone of Tigray region study used Nurses who answer “yes” for the six nursing process implementation questions and observed for their performance, but current study used nurses self-report for implementation.

With regards to the specific components implemented; 71.7% of nurses developed nursing diagnosis, 71% developed care plan, 67.4% implemented the plan, 64.5% evaluated the intervention’s effectiveness and 70.3% documented their nursing activities; which shows inconsistency between the implementation of the five components of the nursing process. It was not inline to study result of Afar region where Assessment and diagnosis was performed by 56.9% of nurses, planning 46%), 38% implemented the plan and 36% evaluated. The difference might be due to difference in sample size and related to the disparity in the overall implementation between the two studies [21].

In this study predictors of implementation of the nursing process were educational level, on-job training, and administrative support. Diploma nurses were less likely to implement the nursing process compared to Bsc nurses (AOR = 0.36, 95% CI (0.14, 0.98). This could be due to varying knowledge and skills during their college and university stay. Nurses working in hospitals that support the nursing process implemented the nursing process 5 times higher than those who did not support NP implementation (AOR = 4.6, 95% CI, (1.87,11.36). This could be because there would be follow-up and supervision, supply of resources, materials, recognition and promotion of those who applied the NP, if the hospitals were to support. Nurses who had nursing training had implemented the NP 4 times higher than untrained nurses (AOR, 3.8, 95% CI, (1.47, 9.94). This could be due to training might have increased their confidence and motivation to enforce the nursing process.

Factors such as organizational structures and facilities, workplace atmosphere, non-proportional nurse to patient ratio / work load, lack of training and motivating factors such as salary, high patient flow and resource shortages are related in other studies. The other is nurse-related factors such as educational level, nursing process knowledge and skills, work experience and the ability to collect needed materials. Certain factors such as severity of the cases and patient engagement impaired nursing process implementation [15, 16, 18, 19].

## Conclusion

The implementation of nursing process was good compared to other studies in Ethiopia where; nearly seven in every ten nurses implemented the nursing process. This study found that educational level, hospital administrative support, and on the job education are predictors of application of the nursing process. Caring is the cornerstone of nursing profession, for nurse to provide appropriate high quality care the nursing process is mandatory and nurses need to go through rigorous academic and clinical preparation on nursing process. Hence, it is proposed that the health minister and Minister of science and higher education should consider the requirement of the nursing process in the professional nursing practice. The health service management, in collaboration with Ethiopian nursing/professional associations and international governmental and non-governmental organizations should give continuous on the job professional development education, and develop nursing practice guidelines. Furthermore, large scale nationwide observational studies should be conducted to track the different factors in the different regions of the country.

## Supplementary Information

**Additional file 1.** Nursing process questionnaire

## Data Availability

The data used and analyzed in this study is available from the corresponding author on reasonable request.

## Abbreviations

AOR: Adjusted odds ratio
Bsc: Bachelor of science
COR: Crude odds ratio
EPI Info: Epidemiological information
IRB: Institutional review board
NP: Nursing process
P: Proportion
SPSS: Statistical package for social science
