# Supplementary material for: Implementation and factors affecting the nursing process among nurses working in selected government hospitals in Southwest Ethiopia
Source: BMC Nurs. 2020 Nov 10;19:105. doi: 10.1186/s12912-020-00498-8 (PMC7654185; doi:10.1186/s12912-020-00498-8)
Supplement: Supplementary file 1 — Additional file 1. Nursing process questionnaire [file 12912_2020_498_MOESM1_ESM.pdf]

Annex-II: Questionnaire

Addis Ababa University College of Health Science

Department of Nursing and Midwifery

Consent Form and information sheet

This questionnaire is prepared to assess the implementation of nursing process and factors affecting implementation of nursing process among nurses working in selected government hospitals in south west Ethiopia. The assessment is made for the partial fulfillment of Masters Degree in Child health Nursing. The results of the study will be used as base line information to design appropriate intervention strategies to increase nurses' capacity to conduct nursing process for their patients. The questionnaire contains both closed and open ended questions and will be provided in self administered form.

The information you provide is confidential and is used only for the purpose of this study. If you have any question, don‘t hesitate to ask the data collector. Your cooperation and participation until the completion of the questionnaire is very necessary for the successful completion of the assessment. We therefore ask your genuine willingness.

Risk/ Discomfort

By participating in this research project, you may feel that it has some discomfort especially on wasting time. But we hope you will participate in the study by considering the benefit of the research result. There is no risk in participating in this research project.

Benefits

If you participate in this research project, there may not be direct benefit to you but your participation is likely to help us in assessing implementation of nursing process among nurses. Ultimately, this will help us to identify the gap and take the appropriate intervention by the authorized stakeholder.

Incentives

You will not be provided any incentives or payment to take part in this project

Right to refuse or withdraw:

You have full right to refuse from participating in this research. You can choose not to respond to some or all questions if you do not want to give your response. You have also the full right to withdraw from this study at any time you wish, without losing any of your right.

Persons to contact:

If you have any question to ask, please contact

Zerihun Adraro

Tel: +251-910-173934

Email = ***adrarozerihun12@gmail.com***

Are you voluntary Yes ☐ No ☐

Thank you in advance for your cooperation

Part I. Socio-demographic data

Instruction;-For closed ended questions circle on your responses and for open ended questions write your response on the spaces provided

s/n	question	response	skip	remark
101	Sex	1. male<br>2. female		
102	Age	-------years		
103	Marital status	1. Single<br>2. Married<br>3. Widowed<br>4. Divorced		
104	Religion	1. orthodox<br>2. protestant<br>3. Muslim<br>4. Catholic<br>5. others		
105	Ethnicity	1. Kafa<br>2. sheka<br>3. Bench<br>4.Amhara<br>5.Oromo<br>6. others specify		
106	Educational status	1. Diploma<br>2. BSc<br>3. MSc		
107	Years of experience	---------years		
108	Institutions from where educational award is obtained	1. Government<br>3. Private		

109	The name of the hospital where you are working in	1.Gebrtsadik shawo<br>2.Mizan aman general hospital<br>3. Teppi hospital		
110	Monthly income in birr	--------Ethiopian birr		

Part II; -Nursing process implementation related questions
Instruction;-For closed ended questions circle on your responses under response column

s/n	question	response	skip	remark
201	Do you follow the steps of nursing process during provision of care?	1. Yes<br>2. No		
202	Have you developed nursing diagnosis from your assessment?	1. Yes<br>2. No		
203	If your answer is yes for Q #202, which of the following did you implement? (More than one answer is possible)	1. Actual<br>2.Risk/potential<br>3. Possible<br>4. Wellness<br>5. Collaborative		
204	Have you been preparing care plan based on your diagnosis?	1.Yes<br>2. No		
205	Have you been implementing the care plan you have developed?	1. Yes<br>2. No		
206	Have you been evaluating the effectiveness of your intervention?	1.Yes<br>2. No		
207	Have you been documenting your nursing care plan?	1.Yes<br>2. No		

Part III - Questions related to factors affecting implementation of NP

Instruction;-For closed ended questions circle on your responses and for open ended questions write your response on the spaces provided under response column.

s/n	question	response	skip	remark
501	Does the hospital administration support the implementation of NP?	1.YES<br>2. NO		
502	Is the allocation of resources for implementation of NP adequate?	1. YES<br>2. NO		
503	Is allocated time sufficient to implement the nursing process?	1.YES<br>2.NO		
504	Is the nurse/patient ratio optimal to implement the nursing process?	1. YES<br>2. NO		
505	Are there monitoring and evaluation for implementation of NP?	1. YES<br>2.NO		
506	Are the salary and promotion motivating for implementation of NP?	1. YES<br>2. NO		
507	Have you ever seen other nurses applying the NP?	1. YES<br>2. NO		
508	Have you got on job training on nursing process?	1. YES<br>2. NO		
509	If your answer is no for Q#508 why?	1.No training is adjusted by the hospital<br>2. I am not willing to train<br>3. Others specify		
510	Is your educational level adequate to implement NP?	1. YES<br>2. NO		
511	If your answer is no for Q # 510, what is the adequate educational level to implement NP?			
